# The importance to update the guidelines for the use of genetic testing in noncancer patients in Brazil

**DOI:** 10.1590/S0034-8910.2015049005988

**Published:** 2015-10-02

**Authors:** Tirzah Braz Petta Lajus

**Affiliations:** I Centro Avançado de Oncologia (Cecan). Liga Contra o Câncer. Departamento de Pesquisa Translacional. Natal, RN, Brasil; II Departamento de Biologia Celular e Genética. Universidade Federal do Rio Grande do Norte. Natal, RN, Brasil

**Keywords:** Supplemental Health, Health Maintenance Organizations, Private Health Care Coverage, Genetic Testing, Genetic Predisposition to Disease, Neoplasms, diagnosis

## Abstract

The Brazilian National Regulatory Agency for Private Health Insurance and Plans has recently published a technical note defining the criteria for the coverage of genetic testing to diagnose hereditary cancer. In this study we show the case of a patient with a breast lesion and an extensive history of cancer referred to a private service of genetic counseling. The patient met both criteria for hereditary breast and colorectal cancer syndrome screening. Her private insurance denied coverage for genetic testing because she lacks current or previous cancer diagnosis. After she appealed by lawsuit, the court was favorable and the test was performed using next-generation sequencing. A deletion of MLH1 exon 8 was found. We highlight the importance to offer genetic testing using multigene analysis for noncancer patients.

## Abstract (pt)

Recentemente a Agência Nacional de Saúde publicou nota técnica que define os critérios para a cobertura de teste genético para diagnóstico de câncer hereditário. Aqui mostramos o caso de uma paciente com nódulo benigno na mama e com extenso histórico de câncer, e que foi encaminhada a um serviço privado de aconselhamento genético. A paciente se enquadra em ambos critérios para rastreamento de síndrome de câncer de mama e colorretal hereditário. O seguro de saúde da paciente negou o pedido de realização do teste genético por ausência de diagnóstico atual ou prévio de câncer. A paciente recorreu judicialmente, o tribunal foi favorável e o teste foi realizado utilizando sequenciamento de próxima geração. A deleção do exon 8 do gene MLH1 foi encontrada. Destacamos a importância de se oferecer testes genéticos pela análise multigene para pacientes sem câncer.

## INTRODUCTION

Since January 2014, the *Agência Nacional de Saúde* (ANS – Brazilian National Regulatory Agency for Private Health Insurance and Plans) has included 22 new guidelines (Anexo da Nota 876/GGRAS/DIPRO/ANS) concerning the utilization of DNA sequencing and of fluorescence in situ hybridization for microduplications and microdeletions to diagnose 29 genetic diseases. Herein we show a report of a patient that did not meet the ANS criteria to benefit from genetic testing, but did meet international guidelines, and was diagnosed with a MLH1 germline mutation. We highlight that the patient inclusion criteria and for molecular methodology of the ANS guidelines should be reviewed.

This article highlights the importance of discussing the criteria for molecular diagnosis in the Brazilian health care system.

## CASE REPORT AND DISCUSSION

A 36-year-old patient was referred to a private genetic counseling service because, besides an extensive family history of cancer, she had a 2.0 cm lump detected in her right breast, as assessed by Magnetic Resonance Imaging, of Breast Imaging Reporting and Data System (BIRADS) category 4. The biopsy by fine needle aspiration showed a ductal proliferative breast lesion. The patient had a high risk for breast cancer and family history of cancer, with kindred diagnosed before the age of 50 years. The analysis of her pedigree showed an autosomal dominant disorder from her paternal side (Figure) with early onset cases of colon adenocarcinoma, gastric, lung, prostate and pancreas cancer.

**Figure:**
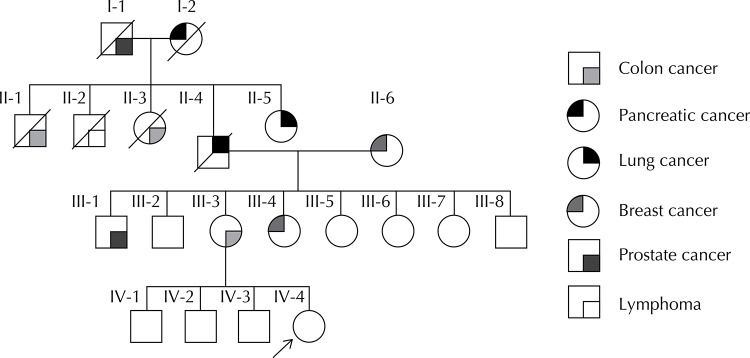
Pedigree of the index patient (indicated by an arrow). Cancer types are described in the figure.

According to the ANS guideline, the patient did not meet the criteria to be tested for germline mutations because (i) she had no current or previous cancer diagnosis, (ii) she was not of Ashkenazi jewish origin, and (iii) there were no known mutations in her family. It is important to highlight that the patient was informed she was not the perfect index patient for family genetic counseling since she was not diagnosed with cancer; thus, her result was limited to individual genetic counseling, i.e., a negative result for presence of mutation would not mean the absence of mutation in her family.

Since Brazil lacks a specific guideline to screen hereditary cancer, the worldwide applied United States National Comprehensive Cancer Network (NCCN) is routinely used. It comprises recommendations on the prevention, diagnosis, and management of malignancies across the continuum of care. The NCCN Guidelines incorporate real-time updates in keeping with the rapid advancements in the field of cancer research and management. For hereditary nonpolyposis colorectal cancer (HNPCC), or Lynch syndrome (LS), the patient should meet revised Amsterdam criteria^8^ or revised Bethesda criteria.^7^

Genetic counseling consultation is a health service that provides information and support to people who have or may be at risk for genetic disorders. It also addresses patients’ specific questions and concerns. The consultation is held by a multidisciplinary team with a psychologist, a medical doctor and a geneticist. After careful medical data collection and considering the patient history, she met NCCN Genetic/Familial High-Risk Assessment criteria both for breast and ovarian and for colorectal cancer, contrary to the ANS guideline. She was considered eligible for genetic screening, and the genes implicated with hereditary breast and ovarian cancer and hereditary nonpolyposis colorectal cancer were chosen to be sequenced.

After the oncologist request for genetic testing, the patient contacted her private insurance and had her request denied because she had no current or previous cancer diagnosis. The patient then appealed the court decision and had the DNA sequencing analysis.

The patient and her family received genetic counseling and written informed consent was obtained. Genomic DNA was extracted from saliva using Oragene DNA (DNA Genotek, Canada). Next-generation DNA sequencing was performed in Illumina MiSeq using TruSight Cancer Sequencing Panel, which targets 94 genes that predispose to several cancers (e.g., breast, colorectal, lung), including rare cancers. In addition, the panel includes 284 single-nucleotide polymorphisms suspected to be associated with cancer by genome-wide association studies. The probe set was designed to enrich for > 1,700 exons, spanning 94 genes of interest, and targets a total of 255 kb of the human genome. The 80-mer probes target libraries of approximately 500 bp (insert size of 300 bp), enriching 350-650 bases centered symmetrically around the midpoint of the probe, with a recommended mean coverage of 100%. This means that the kit covers exonic and noncoding DNA in exon-flanking regions, on average 50 bp. This test analyses both coding regions for genes involved in hereditary breast, ovarian, and nonpolyposis colorectal cancer (ATM, BARD1, BRCA1, BRCA2, BRIP1, CDH1 CHEK2, MLH1, MSH2, MSH6, MUTYH, PALB2, PMS2, PTEN, RAD51C, RAD51D and TP53).

The DNA analysis method registered in the ANS guideline is sequencing of the coding regions by Sanger.^5^ In the Brazilian Hierarchical Classification of Medical Procedures, the code TUSS22 N 40.50.31-00 represents Sanger sequencing of 100 bp per patient, per sample. The gene BRCA1 (Online Mendelian Inheritance in Man [OMIM 113705]) has 81,189 bp, and BRCA2 (OMIM 600185) has 84,193 bp, both totaling 165,382 bp, which means 1,600 times the procedure registered at the aforementioned classification. In Brazil, the cost to sequence the genes BRCA1 and BRCA2 by Sanger is 4,000 BRL (around 1,120 USD), while sequencing 69 genes by NGS costs 6,900 BRL (around 1,630 USD). The difference is negligible for the amount of studied genes. However, it is very important to target the genes of interested that will be analyzed, furthermore when the goal is to diagnose cancer predisposition syndromes for which few guidelines are available.

The result found by next-generation DNA sequencing from the index patient has showed a deletion in MLH1 exon 8 (OMIM 120436). This mutation was not previously described in the National Center for Biotechnology Information (http://www.ncbi.nlm.nih.gov) or in the Leiden Open Variation Database (http://databases.lovd.nl/genomed/home); however, large exon deletion is often classified as pathogenic and related to hereditary nonpolyposis colorectal cancer.

Hereditary nonpolyposis colorectal cancer is a genetically heterogeneous disease subdivided into (1) Lynch syndrome I, or site-specific colonic cancer, and (2) Lynch syndrome II, or extracolonic cancer, particularly carcinoma of the stomach, endometrium, biliary and pancreatic system, and urinary tract.^2^ ^,^ ^4^ About 50.0% from all hereditary nonpolyposis colorectal cancer with identified mutation is associated with MLH1, which codes a protein involved in DNA repair.

Several guideline protocols have been developed to identify families with inherited colorectal cancer and/or Lynch syndrome. The Amsterdam I criteria were initially coined to identify families with colorectal cancer. The finding of extracolonic cancers, especially endometrial cancer, in Lynch syndrome prompted introduction of the revised Amsterdam criteria.^8^ The Bethesda guidelines included testing for tumor marker microsatellite instability (MSI), and the revised Bethesda criteria^7^ specified all cancers known at the time to be associated with the syndrome. Prostate cancer has also been shown to be part of the syndrome.^6^

The clinical testing criteria for hereditary nonpolyposis colorectal cancer currently applied (based on personal and family history) meets revised Bethesda criteria or revised Amsterdam criteria, in which at least three relatives must have had a cancer associated with Lynch syndrome (colorectal, cancer of endometrium, small bowel, ureter or renal-pelvis); all of the following criteria should be present:

- One must be a first-degree relative of the other two;
- At least two successive generations must be affected;
- At least one relative with cancer associated with LS should be diagnosed before the age of 50 years;
- Familial adenomatous polyposis should be excluded in colorectal cancer cases (if any);
- Tumors should be verified whenever possible.

Lynch and de la Chapelle^3^ provided an extensive review of clinical features, pathology, molecular genetics, surveillance, and management in hereditary nonpolyposis colorectal cancer. They emphasized the importance of ascertaining cancer of all anatomic sites as well as noncancer phenotypic stigmata in assessing a family cancer history to allow defining the specific colorectal cancer syndrome concerned. In this first analysis, mutation carriers had an overall 50.4% cumulative risk of developing colorectal cancer by the age of 70 years (54.3% in men and 46.3% in women). The risk for men with MLH1, MSH2, and MSH6 mutations was 57.9%, 53.6%, and 36.2%, respectively, whereas for women it was 50.2%, 47.7%, and 18.3%, respectively.^1^

The molecular diagnosis of hereditary nonpolyposis colorectal cancer in the index patient had an important role in individual and familial follow-up. The patient had been monitored only by a breast surgeon; however, after the result, the gastrointestinal tract was the center of the problem and she looked for a proctologist and gastroenterologist. Her family was aware of the hereditary risk for colorectal cancer and for extracolonic cancers, especially endometrial cancer.

The impact of genetic counseling in this family highlights the importance to track individuals at risk for hereditary cancer, in spite of the patient lacking a previous or current diagnosis of cancer. The main objective of DNA sequencing to identify germline mutations is to offer early detection of cancer; nevertheless, in some cases, prophylactic surgery should be recommended. The earliest the cancer diagnosis is concluded, the better is the patient prognosis.

Furthermore, early diagnosis can reduce treatment costs. For follow-up screening, this patient will undergo annual mammogram, breast ultrasound or magnetic resonance imaging, and colonoscopy, but certainly the costs for these exams are lower than those for cancer treatment, which can be around 10,000 BRL (around 2,850 USD) per month for Herceptin (www.brasindice.com.br).

In conclusion, this case report highlights the importance of performing multigene analysis by next-generation DNA sequencing in families with cancers at multiple sites, which reinforces the need to update the ANS guideline in Brazil. Herein, the patient’s needs and concerns had changed after the molecular diagnosis, since the family was concerned only about breast annual exams, and thereafter they were encouraged to be followed also by a gastroenterologist. The molecular diagnosis by deep sequencing gave the opportunity for genetic counseling and thus general medical awareness will be performed.
